# Significant enhancement in the rate of body mass and lean body mass gains with supplementation of a bio-active peptide in conjunction with eight weeks of resistance training: a prospective, double-blind, placebo controlled randomized trial

**DOI:** 10.1186/1550-2783-12-S1-P47

**Published:** 2015-09-21

**Authors:** Patrick L Jacobs

**Affiliations:** 1 Superior Performance Research, LLC; Miami, FL 33187, USA

## Background

It has been previously shown that supplementation with a commercial food-based bio-active peptide product (Bio-Gro™) may improve resistance training capacity and enhance recovery between repeated bouts of strenuous exercise. It has been suggested that enhanced workout capacity during training sessions and improved recovery from exercise may enhance chronic training adaptions, such as superior gains in muscular mass, compared with training without supplementation. The purpose of the present investigation was to examine the changes in body mass and lean body mass of young men engaging in eight weeks of resistance training and ingesting a commercial bio-active product compared with training without supplementation.

## Methods

This study utilized a prospective, randomized, double-blind, placebo-controlled research design. Twenty recreationally resistance trained men voluntarily participated in this study. Each research participant agreed to participate in four intense weight training sessions per week for four weeks and five intense weight training sessions per week for weeks five through eight over an eight week study period. Study participants were randomly assigned to receive either Bio-Gro™ or placebo for the eight week period and were directed to take two servings per day. Test sessions were performed prior to initiation of the study and following the eight weeks of training. Body composition was assessed using the BodPod system, which utilizes air displacement. Body composition was also calculated based on skinfold measurements that were taken at the chest, abdomen, thigh, triceps, and suprailiac. Circumferential measurements were taken at standard sites using a Gulick tape and included chest, shoulders, abdomen, mid-thigh, mid-arm relaxed, and mid-arm flexed. Pre- and post-study measurements were used to establish change scores which were compared between the Bio-Gro™ and placebo groups using one way ANOVAs. Statistical significance was accepted at the p < 0.05 level.

## Results

Analyses revealed no significant differences between groups in baseline measures of body composition or circumferential measures (p's > 0.05). Analyses of change scores between groups indicated that Bio-Gro™ produced significantly greater (p < 0.05) changes in total body mass as assessed with BodPod (+6.3 pounds) than the placebo condition (+2.8 pounds). Lean body mass changes were also significantly greater with Bio-Gro™ (+5.8 pounds) compared with placebo (+3.7 pounds) (p < 0.05).

While there were significant main effects of time detected for change scores of total body mass and lean body mass as calculated from the skinfold measurements, there were no significant group × time interactions indicating no significant differences in changes scores between groups.

Analyses of change scores between groups indicated that Bio-Gro™ produced significantly greater changes in mid-arm flexed measurements (+0.74 inches) than the placebo condition (+0.31 inches) (p < 0.05). There were no other significant differences detected between groups in circumferential change scores.

## Conclusion

The findings of this prospective, randomized, double-blind, placebo-controlled research investigation indicate that when applied in conjunction with an intense eight week resistance training program, Bio-Gro™, a bio-active peptide supplement, produced significantly greater gains in total body mass (125% greater) and significantly greater increases in lean body mass compared with placebo with significantly greater changes in flexed mid-arm circumference (p < 0.05).

